# Mood and Activity Measured Using Smartphones in Unipolar Depressive Disorder

**DOI:** 10.3389/fpsyt.2021.701360

**Published:** 2021-07-09

**Authors:** Morten Lindbjerg Tønning, Maria Faurholt-Jepsen, Mads Frost, Jakob Eyvind Bardram, Lars Vedel Kessing

**Affiliations:** ^1^Copenhagen Affective Disorder Research Center (CADIC), Psychiatric Center Copenhagen, Copenhagen, Denmark; ^2^Department of Clinical Medicine, Faculty of Health and Medical Sciences, University of Copenhagen, Copenhagen, Denmark; ^3^Monsenso A/S, Copenhagen, Denmark; ^4^Copenhagen Center for Health Technology, Copenhagen, Denmark; ^5^Department of Health Technology, Technical University of Denmark, Lyngby, Denmark

**Keywords:** depression, unipolar depressive disorder, smartphone, technology, ecological momentary assessments, mHealth

## Abstract

**Background:** Smartphones comprise a promising tool for symptom monitoring in patients with unipolar depressive disorder (UD) collected as either patient-reportings or possibly as automatically generated smartphone data. However, only limited research has been conducted in clinical populations. We investigated the association between smartphone-collected monitoring data and validated psychiatric ratings and questionnaires in a well-characterized clinical sample of patients diagnosed with UD.

**Methods:** Smartphone data, clinical ratings, and questionnaires from patients with UD were collected 6 months following discharge from psychiatric hospitalization as part of a randomized controlled study. Smartphone data were collected daily, and clinical ratings (i.e., *Hamilton Depression Rating Scale 17-item*) were conducted three times during the study. We investigated associations between (1) smartphone-based patient-reported mood and activity and clinical ratings and questionnaires; (2) automatically generated smartphone data resembling physical activity, social activity, and phone usage and clinical ratings; and (3) automatically generated smartphone data and same-day smartphone-based patient-reported mood and activity.

**Results:** A total of 74 patients provided 11,368 days of smartphone data, 196 ratings, and 147 questionnaires. We found that: (1) patient-reported mood and activity were associated with clinical ratings and questionnaires (*p* < 0.001), so that higher symptom scores were associated with lower patient-reported mood and activity, (2) Out of 30 investigated associations on automatically generated data and clinical ratings of depression, only four showed statistical significance. Further, lower psychosocial functioning was associated with fewer daily steps (*p* = 0.036) and increased number of incoming (*p* = 0.032), outgoing (*p* = 0.015) and missed calls (*p* = 0.007), and longer phone calls (*p* = 0.012); (3) Out of 20 investigated associations between automatically generated data and daily patient-reported mood and activity, 12 showed statistical significance. For example, lower patient-reported activity was associated with fewer daily steps, shorter distance traveled, increased incoming and missed calls, and increased screen-time.

**Conclusion:** Smartphone-based self-monitoring is feasible and associated with clinical ratings in UD. Some automatically generated data on behavior may reflect clinical features and psychosocial functioning, but these should be more clearly identified in future studies, potentially combining patient-reported and smartphone-generated data.

## Introduction

Unipolar depressive disorder is a common and serious mental disease with a lifetime prevalence of 15–20% (1) and is a leading cause of disability and disease worldwide (2). Depressive episodes are associated with changes in mood and energy (3) as well as behavioral components such as changes in activity level (4), psychomotor function (5), and social interactions (6) – all of which are likely collectible through smartphones.

Currently, no objective biomarkers are available to monitor illness activity in patients with unipolar depressive disorder. Monitoring of symptoms is essential for patients and clinicians, i.e., as part of measurement-based care (7). Further, it allows researchers to gain novel insights into psychopathology and evaluate the effectiveness of interventions. Traditionally symptom monitoring relies on clinical evaluation and questionnaires with a risk of recall bias (8) or patients backfilling data on the day of the visit (9) and does not capture daily fluctuations, although, they might be clinically important (10).

Thus, monitoring methods to assess patients regularly, on both subjective changes in mood and detectable changes in behavior and psychomotor function, are warranted. Remote monitoring of patients could potentially help to allocate the right treatment to the right patient at the right time to distribute the limited treatment resources appropriately and possibly detect relapse in high-risk groups.

Smartphones comprise an available platform for remote real-time monitoring of patient-reported symptoms such as mood, activity, and anxiety through Ecological Momentary Assessments (EMAs) (11, 12). Further, data generated automatically from sensors and logs, such as the number of steps, ingoing and outgoing calls, ingoing and outgoing text messages, or location information, might reflect changes in behavior and psychomotor function (13–15), and possibly even allow for digital phenotyping (16).

Patient reportings are often easier to interpret; however, they require the patient's active action to provide data. Automatically generated data allow for large-scale data collection without efforts to the patients, but often with several technical challenges and more complex interpretation.

Several relevant reviews within the field (11, 17–21) indicate that smartphone-based symptom monitoring is feasible. Both in terms of automatically generated data and patient-reportings, i.e., increased screen time seems to be associated with higher levels of depression, along with more incoming calls and longer call duration (21). However, most available studies include participants with depressive symptoms without diagnostic evaluation or participants with other psychiatric diagnoses. Only a few relevant studies regarding smartphone-based symptom-monitoring in patients with a diagnosis of a depressive disorder have been published (22–24): Thus, very limited research has been done combining smartphone-based EMA and classical psychometrics in patients diagnosed with unipolar depressive disorder. Without this link, the utility of smartphone-based interventions and monitoring tools will be difficult to translate and implement into clinical praxis. Important work on smartphone-based EMA's in clinically well-characterized populations has been done in patients with schizophrenia spectrum disorders (25, 26) and bipolar disorder (14, 27–30) with promising results.

The present study aims to add important knowledge to the field by (1) investigating associations between daily smartphone-based patient-reported mood and activity and clinical ratings of depression measured using the Hamilton Depression Rating Scale 17-items (HDRS-17), psychosocial functioning measured using the Functional Assessment Short Test (FAST), and standardized questionnaires using Beck's Depressive Inventory (BDI-21); (2) investigating associations between automatically generated smartphone measures of social and physical activity as well as phone usage with validated clinical ratings (the HDRS-17 and the FAST) and (3) investigating associations between automatically generated smartphone data of social and physical activity as well as phone usage with same-day smartphone-based patient-reportings of mood and activity.

Based on available studies and clinical experience, we hypothesized that (1) higher symptom scores on the HDRS-17, the FAST, and the BDI-21 would entail lower smartphone-based patient-reported mood and activity; (2) higher symptom scores on the HDRS-17 and FAST would entail lower physical and social activity and higher smartphone phone usage, measured by automatically generated smartphone data, and (3) lower smartphone-based patient-reported mood and activity would entail lower physical and social activity and higher phone usage measured by automatically generate smartphone data.

## Materials and Methods

The data included in the present study were collected as part of a large Randomized Controlled Trial (RCT) called the RADMIS trial, investigating the effect of smartphone-based monitoring and treatment in patients with unipolar depressive disorder following discharge from a psychiatric hospital. The results from the RCT study have been published elsewhere (31).

The RADMIS trial included 120 patients diagnosed with unipolar depressive disorder when discharged from psychiatric hospitals in The Capital Region in Denmark from May 2017 to August 2019. The present study includes data from 74 of these patients, as they provided relevant smartphone data for the analysis.

**Inclusion criteria:** Age over 18 years; unipolar depressive disorder diagnosis according to the International Classification of Diseases, version 10 (ICD-10) using Schedules for Clinical Assessments in Neuropsychiatry (SCAN) (32) (ICD-10 codes: 32.0–33.31); discharge from a psychiatric hospital following hospitalization for a depressive episode.

**Exclusion criteria:** Pregnancy; insufficient Danish language skills.

All patients were initially diagnosed by the clinicians in the wards. Subsequently, the diagnosis was confirmed by a SCAN interview conducted by SCAN-certified medical doctors with access to the patients' electronic medical records. Patients were thoroughly assessed, and few exclusion criteria were applied to resemble the clinical population of patients needing hospitalization due to their depression.

In brief, patients in the RADMIS trial were randomized 1:1 to either the intervention group or the control group. The intervention group received a smartphone-based monitoring and treatment system [the Monsenso system (33)] that allowed patients to self-monitor various symptoms such as mood, sleep, and activity on a daily basis and further collected various automatically generated smartphone data from smartphone sensors and logs. The data was displayed graphically in the app to the patient and made available to a study nurse who, based on the smartphone data, guided and supported the patient during the 6 months following discharge. The control group had the smartphone app installed for the collection of automatically generated smartphone data, (i.e., for use in the present study) but without access to any content or support. The trial lasted 6 months following discharge from psychiatric hospitalization. For further information, see original publications from the RADMIS trial (31, 34).

### Data Collection

At 0, 3, and 6 months following inclusion in the study, patients were assessed and rated by research-trained medical doctors and filled in paper-based questionnaires. In addition, numerous data was continuously collected via the smartphone during the study period:

#### Clinical Ratings

The severity of depressive symptoms for the past 3 days was measured using the HDRS-17 (35). The total score is between 0 and 52, with the following cutoff scores: 13–17 mild depression 18–24 moderate depression, and 25–52 severe depression (36). Further, we used sub-item 1 addressing decreased mood (0–4), sub-item 8 addressing psychomotor retardation (0–4), and sub-item 9 addressing psychomotor agitation (0–4).

The level of psychosocial functioning for the past 2 weeks was measured using the FAST (37). The total score is between 0 and 72, with higher scores resembling lower psychosocial functioning. The scale measures the following domains: Autonomy, work-function, cognitive functions, financial issues, interpersonal relations, and leisure activities. Each domain contains several items rated between 0 (no difficulties) and 3 (severe difficulties).

#### Questionnaires

Self-reported depressive symptoms were measured using BDI-21 (38). The total score is between 0 and 63, with the following cutoff scores: 10–18: mild-moderate depression; 19–29: moderate-severe depression; ≥30: severe depression.

#### Smartphone Data

*Smartphone-based patient-reportings:* The patients in the original intervention group reported symptoms daily using the smartphone app. Mood was scored with a choice between five mood scores: −3 (severe depression), −2 (moderate depression), −1 (mild depression), −0.5 (lowered mood), 0 neutral mood). Activity was scored as one of the following scores: −3, −2, −1, 0, 1, 2, 3 with negative values resembling low activity; zero is the patient's normal activity level, and positive score resembling higher than usual activity. Once a day, at a self-chosen time, the smartphone reminded the patient to conduct the evaluations. If several days were missing, patients were contacted by the study nurse.

*Automatically generated smartphone data:* The automatically generated smartphone data were collected from patients in both the intervention and control groups. The smartphone app was available for Android and iOS. Due to technical constraints on iOS, fewer automatically generated data were available from iOS users (i.e., we did not have access to sensor data on iOS, and early iOS users did not provide any automatically generated data). The automatically generated smartphone data were collected and summarized to daily measures and reflected physical activity, social activity, and phone usage:

*Physical activity:* The number of steps per day; The total distance moved per day (based on the Global Positioning System (GPS), Wifi signals, and mobile cell towers).

*Smartphone usage:* The total screen-on time per day (seconds per day) and the number of times the screen was turned on per day.

*Social activity:* The number of incoming, outgoing, and missed calls per day; the duration of calls per day (seconds per day); The number of incoming and outgoing text messages per day (not including text message applications or social media).

No specific instructions on how and when to carry the phone were given.

### Ethical Considerations

Ethical permission was obtained from the ethics committee in The Capital Region of Denmark (H-16046093) and the Data Agency (RHP-2017-005, I-Suite: 05365). The law on handling personal data, as well as the European General Data Protection Regulation (GDPR), was respected. All data collected by researchers was stored in the Research Electronic Data Capture (REDCap) electronic data capture tools (39, 40). Electronic data from the Monsenso app was stored by Monsenso with a data storage agreement between Monsenso and The Capital Region of Copenhagen. All patients were given written and oral information and gave informed consent, according to the Helsinki declaration.

### Statistical Analyses

The statistical analyses for the present study were defined a priori. For aims 1 and 2, we calculated the averages of the daily smartphone data (automatically generated data as well as patient-reportings) for the days surrounding the ratings and questionnaires. We used the day of the rating/questionnaire and, respectively, 3 days before the HDRS-17 ratings and BDI-21 questionnaires and 14 days before the FAST ratings. This resembles the period in which the symptoms were evaluated. In a few cases, relevant smartphone data were not available in the 3/14 days before an assessment. In such cases, we used the 3/14 following days as a pragmatic solution to use the valuable data surrounding the clinical data points. Missing items from ratings and questionnaires were not included in the summed scores, and no imputations or assumptions on missing items were made.

For aim 3, we used all smartphone-based patient-reported data with same-day corresponding automatically generated smartphone-based data without any averages or summed scores.

Linear mixed-effects models accounting for repeated measurements within each participant were employed. For aims 1 and 2, we used averages of smartphone data as the dependent variable and the clinical ratings/questionnaires as independent variables handled as fixed factors. For aim 3, we used automatically generated smartphone data as the dependent variable and patient-reported data as the independent variable handled as fixed factors. Individual ID number was used as a random factor for all analyses to account for individual differences.

All analyses were conducted first in an unadjusted model and secondly in models adjusted for age and sex. As there were only minor differences among adjusted and unadjusted models, only the models adjusted for age and sex are presented. Model assumptions with analyses of residuals and covariance were calculated and assessed graphically (i.e., normality distribution of residuals) and discussed by MFJ and MLT for all models and were acceptable. Further, all automatically generated smartphone variables were logarithm transformed and square-root transformed and included in all analyses without significant improvement of models and therefore omitted. All models had sufficient amount of data to fulfill the model assumptions.

The sample size was defined according to the RCT. As this is the first study on smartphone data in this well-characterized and severely ill group, it is explorative by nature. Therefore, in the present study, the statistical models were not corrected for multiple analyses. Thus, results should be interpreted with caution due to the risk of chance findings when multiple analyses are conducted. *P*-values of ≤0.05 were considered statistically significant. We used SPSS (Statistical Package for the Social Sciences) version 25 for all analyses.

## Results

**Figure 1:**
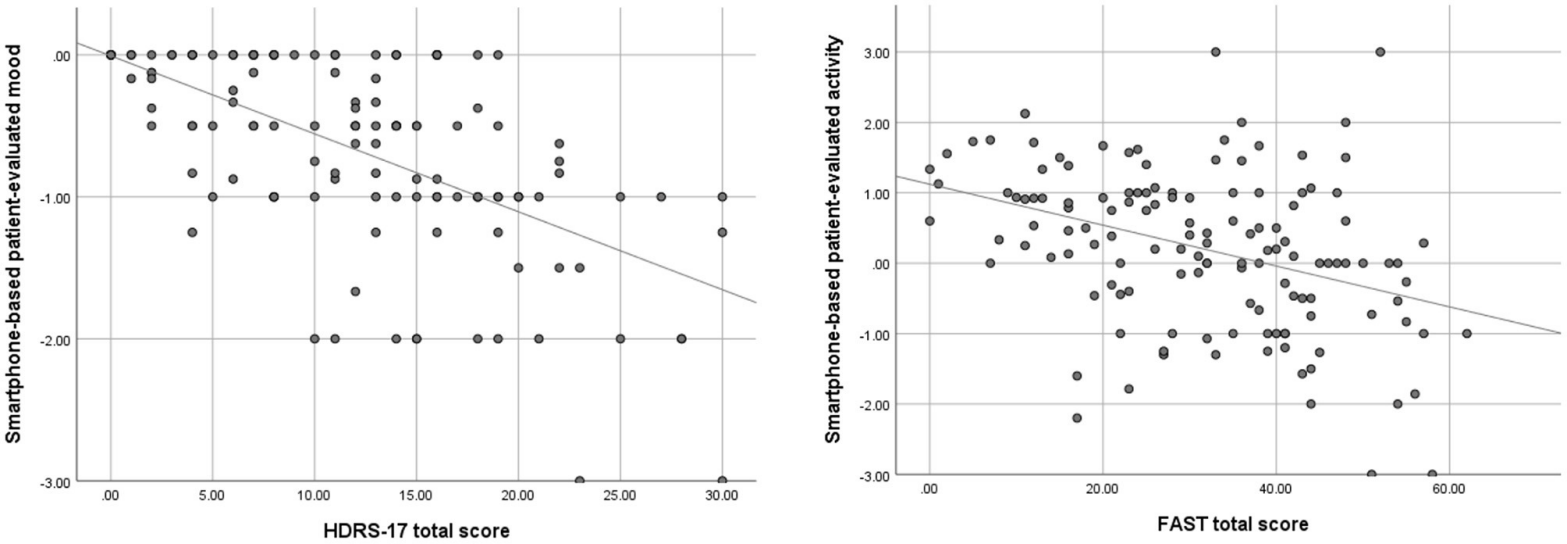
Simple scatterplots, displaying the association between patient-reported mood (average of preceding 3 days) and total scores of the Hamilton Depression Rating Scale 17-items (HDRS-17) as well as patient-reported activity (average of preceding 14 days) and total scores of the Functional Assessment Short Test (FAST), with corresponding simple regression lines.

A total of 74 patients provided data to the present study, with 11,368 days of available smartphone data *(average 158 days/patient, SD* = *68, range 7–388)*. Patient-reported data was provided by 58 patients for 7,509 days (*average 130 days/patient; SD* = *65; range 7–348*), automatically generated smartphone data was provided by 46 patients for 7,063 days *(average of 158 days/patient; SD* = *67; range: 27–384)*, and 30 patients provided 3,204 days of same-day smartphone-based patient-reported data and automatically generated smartphone data *(average 107 days/patient SD* = *52; range 20–171*). The 74 patients participated in 196 clinical ratings (*average 2.6 ratings/patient; range 1–3*) and completed 147 questionnaires *(average 2.0 questionnaires/patient; range 1–3)*. The amount of data included in the individual analyses varies depending on available data and the temporal context of clinical ratings and smartphone data. This information is presented in the corresponding tables. The data collected had a large variance for both smartphone-based patient-reported symptoms and clinical ratings, including cases of both mild, moderate, and severe symptoms, although, fewer cases of severe symptoms. Data examples are displayed in Figure 1.

Sociodemographic and clinical characteristics are presented in Table 1. All patients were diagnosed with moderate-severe depression during the hospitalization leading to the inclusion. The sample thus composed of a population of severely ill patients with the need for hospital care. A total of 29 patients used iPhones, and the remaining used Android phones.

**Table 1:** Sociodemographic and clinical characteristics of all patients providing data (*n* = 74).

**Sociodemographic data**	
Age, years	44.4 (14.5)
Female sex, % (*n*)	52.7 (39)
Years of education after primary school	5.0 (2.8)
Completed highschool, % (*n*)	58.1 (43)
University degree, % (*n*)	36.5 (27)
Unemployed or disability pension	44.6 (33)
In a relationship, % (*n*)	47.3 (35)
Living alone, % (*n*)	50 (37)
**Smartphone usage**	
iPhone, % (*n*)	39.2 (29)
Years with smartphone	5.6 (2.5)
Provided patient-reported smartphone data (original intervention group)	78.4 (58)
**Clinical history**	
Psychiatric comorbidity, % (*n*)	39.2 (29)
Hamilton depression rating scale 17 total score	12 (6–16)
Substance abuse, % (*n*)	13.5 (10)
Age at the first depressive episode, years	33.5 (15.2)
Age at first hospitalization, yeas	40.3 (14.3)
Receiving antidepressant medication at inclusion	97.3 (72)
Depressive episodes, number	3 [2–5]
Previous psychiatric hospitalizations, number	2 [1–3]

*Data are mean (S.D.), median [IQR], or % (n) unless otherwise stated*.

### Patient-Reported Smartphone-Based Data (Aim 1)

Data on the association between smartphone-based patient-reported data on mood and activity with clinical ratings and questionnaires are presented in Table 2 (model adjusted for age and sex).

**Table 2:** Associations between smartphone-based patient-reported mood and activity and observer-based rating scales and questionnaires in 58 patients with unipolar depressive disorder.

	**Adjusted for age and sex**		
	**B**	**95% CI**	***p***
**Smartphone-based patient-reported mood (*****n*** **=** **127)**			
**HDRS-17 total^a^**	**−0.057**	**−0.071;** **−0.043**	** <0.001**
**HDRS sub-item 1^b^**	**−0.44**	**−0.53;** **−0.35**	** <0.001**
**BDI-21^c^**	**−0.034**	**−0.044; 0.024**	** <0.001**
**FAST^d^**	**−0.013**	**−0.019;** **−0.0063**	** <0.001**
**Smartphone-based patient-reported activity (*****n*** **=** **126)**			
**HDRS-17 total^a^**	**−0.053**	**−0.084;** **−0.022**	**0.001**
HDRS-17 sub-item 8^e^	−0.20	−0.44; 0.033	0.091
HDRS-17 sub-item 9^f^	−0.27	−0.60; 0.069	0.12
**FAST^d^**	**−0.022**	**−0.034;** **−0.010**	** <0.001**

a *HDRS-17: The Hamilton Depression Rating Scale 17 total score*.

b *HDRS Sub-item 1 – level of decreased mood*.

c *Beck's Depressive Inventory (BDI-21)*.

d *FAST, The Functional Assessment Short Test total score*.

e *HDRS Sub-item 8 – level of psychomotor retardation*.

f *HDRS Sub-item 9 – level of psychomotor agitation*.

*n = number of clinical ratings with corresponding relevant smartphone data included in the analyses*.

*Bold text reflects statistically significant findings with p < 0.05*.

#### Smartphone-Based Patient-Reported Mood

As hypothesized, smartphone-based patient-reported mood was negatively associated with severity of symptoms in clinical ratings and questionnaires: Higher scores on the HDRS-17, the FAST and the BDI-21 was associated with lower smartphone-based patient-reported mood: [adjusted models: HDRS-17: B = −0.057, 95% CI (−0.071; −0.043), *p* < 0.001; HDRS item 1: B = −0.44, 95% CI (−0.53; −0.35), *p* < 0.001; FAST: B = −0.013, 95% CI (−0.019; −0.0063), *p* < 0.001; BDI-21: B = −0.034, 95% CI (−0.044; 0.024), *p* < 0.001]. Thus, for every increase in the HDRS-17 score of one point there was decreased in smartphone-based patient-reported mood of 0.057 points.

#### Smartphone-Based Patient-Reported Activity

As hypothesized, smartphone-based patient-reported activity were negatively associated with severity of symptoms in clinical ratings: Higher severity of symptoms scores on the HDRS-17 and the FAST were associated with lower smartphone-based patient-reported activity: [adjusted models: HDRS-17:B = −0.053, 95% CI (−0.084; −0.022), *p* = 0.001; FAST: B = −0.022, 95% CI (−0.034; −0.010), *p* = <0.001]. Thus, for every increase in the HDRS-17 score of one point, there was a decreased smartphone-based patient-reported activity of 0.053 points.

We found no statistically significant association between the HDRS subitem 8 (psychomotor retardation) and 9 (psychomotor agitation), respectively, and smartphone-based patient-reported activity.

### Automatically Generated Smartphone Data (Aim 2)

Data on the association between automatically generated smartphone data and clinical ratings are presented in Table 3 (model adjusted for age and sex).

**Table 3:** Associations between automatically generated smartphone data reflecting physical activity, social activity, and phone usage and observer-based clinical ratings in 46 patients with unipolar depressive disorder.

			**Model adjusted for age and sex**	
		**B**	**95%**	***P***
Step count (number/day)^a^ *n* = 47	HDRS-17 total score^b^	−81.24	−168.06; 5.58	0.066
	HDRS sub-item 8**^c^**	−440.78	−1066.07; 184.51	0.161
	HDRS sub-item 9**^d^**	−780.01	1693.81; 133.79	0.092
	**FAST^e^**	**−50.83**	**−98.31;** **−3.36**	**0.036**
Distance travels, location data (meter/day)^a^ *n* = 40	HDRS-17 total score^b^	−891.50	−2336.56; 553.55	0.218
	HDRS sub-item 8**^c^**	−3465.37	−13128.76; 6198.02	0.471
	HDRS sub-item 9**^d^**	5379.38	−9797.46; 20556.22	0.476
	FAST**^e^**	−304.30	−882.39; 273.79	0.294
Screen time (seconds/day)^a^ *n* = 81	HDRS-17 total score^b^	−148.81	−565.08; 267.45	0.479
	HDRS sub-item 8**^c^**	−2131.92	−4911.72; 647.87	0.131
	HDRS sub-item 9**^d^**	−1579.06	−5719.49; 2561.37	0.450
	FAST**^e^**	−62.52	−244.83; 119.79	0.497
Screen on (number/day)^a^ *n* = 81	**HDRS-17 total score^b^**	**−0.71**	**−1.35;** **−0.064**	**0.032**
	HDRS sub-item 8**^c^**	−2.10	−6.48; 2.29	0.341
	HDRS sub-item 9**^d^**	−1.39	−8.53; 5.76	0.699
	FAST**^e^**	−0.12	−0.51; 0.27	0.546
Incoming calls (number/day)^a^ *n* = 89	HDRS-17 total score^b^	0.031	−0.062; 0.12	0.514
	HDRS sub-item 8**^c^**	−0.067	−0.58; 0.44	0.792
	HDRS sub-item 9**^d^**	0.074	−0.71; 0.86	0.852
	**FAST^e^**	**0.042**	**0.0037; 0.081**	**0.032**
Outgoing calls (number/day)^a^ *n* = 89	HDRS-17 total score^b^	0.11	−0.020; 0.25	0.094
	HDRS sub-item 8**^c^**	0.14	−0.75; 1.02	0.759
	**HDRS sub-item 9^d^**	**1.64**	**0.23; 3.05**	**0.023**
	**FAST^e^**	**0.077**	**0.015; 0.14**	**0.015**
Missed calls (number/day)^a^ *n* = 89	HDRS-17- total score^b^	0.039	−0.016; 0.094	0.160
	HDRS sub-item 8**^c^**	−0.00012	−0.36; 0.36	0.999
	HDRS sub-item 9**^d^**	0.32	−0.22; 0.87	0.244
	**FAST^e^**	**0.030**	**0.0086; 0.052**	**0.007**
Duration of phone calls (seconds/day)^a^ *n* = 89	**HDRS-17 total score^b^**	**76.8**	**15.91; 137.69**	**0.014**
	HDRS sub-item 8**^c^**	−46.99	−415.02; 321.04	0.8
	**HDRS sub-item 9^d^**	**666.67**	**122.9; 1210.44**	**0.017**
	**FAST^e^**	**35.64**	**7.96; 63.32**	**0.012**
Incoming text messages (number/day)^a^ *n* = 88	HDRS-17 total score^b^	0.059	−0.14; 0.26	0.561
	HDRS sub-item 8**^c^**	0.63	−0.70; 1.97	0.347
	HDRS sub-item 9**^d^**	0.95	−1.00; 2.90	0.334
	FAST**^e^**	0.067	−0.019; 0.15	0.125
Outgoing text messages (number/day)^a^ *n* = 88	HDRS-17 total score^b^	0.035	−0.11; 0.18	0.632
	HDRS sub-item 8**^c^**	0.27	−0.71; 1.25	0.589
	HDRS sub-item 9**^d^**	0.74	−0.68; 2.16	0.301
	FAST**^e^**	0.043	−0.020; 0.11	0.175

a *Averages of automatically generated smartphone data calculated for the current day and 3 days before ratings with the HDRS and 14 days prior for the FAST rating*.

b *Hamilton Depression Rating Scale (HDRS) 17-item*.

c *HDRS sub-item 8 – level of psychomotor retardation*.

d *HDRS sub-item 9 – level of psychomotor agitation*.

e *FAST: The Functional Assessment Short Test total score*.

*n = number of clinical ratings with corresponding relevant smartphone data included in the analyses*.

*Bold text reflects statistically significant findings with p < 0.05*.

#### Physical Activity

As hypothesized, higher scores on the FAST was associate with lower daily step count: [adjusted model: B = −50.83, 95% CI (−98.31; −3.36) *p* = 0.036]. Thus, for every increase in the FAST of one point, there was a decreased number of daily steps of 51. There were no other statistically significant results concerning step counts or total distance traveled as proxy measures for physical activity.

#### Smartphone Usage

Higher scores on the HDRS-17 were associated with fewer times the screen was turned on: [adjusted model: B = −0.71, 95% CI (−1.35; −0.064), *p* = 0.032]. Thus, for every increase in the HDRS-17 of one point, there was a decrease in the number of times the patient turned on their smartphone screen per day by 0.71. There were no other statistically significant results on the daily number of times the screen was turned on or the screen-on duration, as proxy measures for smartphone usage.

#### Social Activity

Higher scores on the HDRS-17 and the FAST were positively associated with the following measures of social activity (adjusted models): The number of incoming calls was associated with scores on the FAST: B = 0.042, 95% CI (0.0037; 0.081), *p* = 0.032. The number of outgoing calls were associated with scores on the HDRS item 9: B = 1.64, 95% CI (0.23; 3.05), *p* = 0.023 and the FAST: B = 0.077, 95% CI (0.015; 0.14), *p* = 0.015. The number of missed calls were associated with scores on the FAST: B = 0.030, 95% CI (0.0086; 0.052), *p* = 0.007. The duration of phone calls were associated with scores on the HDRS-17: B = 76.8, 95% CI (15.91; 137.69), *p* = 0.014; HDRS item 9: B = 666.67, 95% CI (122.9; 1210.44), *p* = 0.017; and the FAST: B = 35.64, 95% CI (7.96; 63.32), *p* = 0.012. Thus, for every increase in the HDRS-17 of one point, the total duration of daily phone calls increased by 77 s. There were no further statistically significant results on the number of incoming, outgoing, or missed calls, call duration, or the number of incoming or outgoing text messages as proxy measures for social activity.

Overall from aims 1 and 2: A moderately depressed patient with a score of 20 on the HDRS-17 would turn the smartphone screen on 14 times less, have 26 min longer phone conversation as well as a 1.1 lower self-reported mood and activity, compared with a patient of the same age and sex and an HDRS-17 score of 0. A patient with a score of 40 on the FAST would walk 1.020 steps less, have 1.7 more incoming calls, 3.1 more outgoing calls, 1.2 more missed calls, and have 24 min longer duration of phone calls every day. Further, this patient would report 0.5 lower mood and 0.9 lower activity compared with a patient of the same age and sex and a FAST score of 0.

### Smartphone-Based Patient-Reported Mood and Activity Compared With Automatically Generated Smartphone Data (Aim 3)

Associations between smartphone-based patient-reported mood and activity with same-day automatically generated smartphone data are presented in Table 4 (models adjusted for age and sex).

**Table 4:** Associations between automatically generated smartphone data reflecting physical and social activity as well as phone usage and smartphone-based patient-reported mood and activity in 30 patients with unipolar depressive disorder.

	**Model adjusted for age and sex**		
	**B**	**95% CI**	***p***
**Step count (number/day) (*****n*** **=** **17, o** **=** **1,478)**			
Smartphone-based patient-reported activity^a^	**178.58**	**32.61; 324.54**	**0.017**
Smartphone-based patient-reported mood^b^	283.78	−64.01; 631.56	0.11
**Distance traveled (meters/day) (*****n*** **=** **23, o** **=** **947)**			
Smartphone-based patient-reported activity^a^	**4948.64**	**2239.70; 7647.58**	** <0.001**
Smartphone-based patient-reported mood^b^	4933.89	−1873.46; 11741.24	0.16
**Screen time (seconds/day) (*****n*** **=** **30, o** **=** **2,441)**			
Smartphone-based patient-reported activity^a^	**−783.63**	**−1122.90; 444.36**	** <0.001**
Smartphone-based patient-reported mood^b^	**−1343.60**	**−2196.93;** **−490.26**	**0.0020**
**Screen on (number/day) (*****n*** **=** **30, o** **=** **2,441)**			
Smartphone-based patient-reported activity^a^	**1.32**	**0.45; 2.19**	**0.0030**
Smartphone-based patient-reported mood^b^	0.75	−1.49; 3.00	0.51
**Call duration (seconds/day) (*****n*** **=** **30, o** **=** **2,552)**			
Smartphone-based patient-reported activity^a^	−16.24	−63.50; 30.03	0.50
Smartphone-based patient-reported mood^b^	**−256.81**	**−370.13;** **−143.50**	** <0.001**
**Incoming calls (number/day) (*****n*** **=** **30, o** **=** **2,552)**			
Smartphone-based patient-reported activity^a^	**−0.088**	**−0.15;** **−0.023**	**0.0082**
Smartphone-based patient-reported mood^b^	**−0.30**	**−0.46;** **−0.14**	** <0.001**
**Outgoing calls (number/day) (*****n*** **=** **30, o** **=** **2,552)**			
Smartphone-based patient-reported activity^a^	0.042	−0.088; 0.17	0.526
Smartphone-based patient-reported mood^b^	−0.26	−0.57; 0.043	0.093
**Missed calls (number/day) (*****n*** **=** **30, o** **=** **2,552)**			
Smartphone-based patient-reported activity^a^	**0.085**	**0.027; 0.14**	**0.0041**
Smartphone-based patient-reported mood^b^	**−0.17**	**−0.31;** **−0.028**	**0.019**
**Incoming text-messages (number/day) (*****n*** **=** **30, o** **=** **2,453)**			
Smartphone-based patient-reported activity^a^	−0.049	−0.32; 0.22	0.72
Smartphone-based patient-reported mood^b^	**−1.87**	**−2.54;** **−1.20**	** <0.001**
**Outgoing text-messages (number/day) (*****n*** **=** **30, o** **=** **2,453)**			
Smartphone-based patient-reported activity^a^	−0.17	−0.42; 0.088	0.20
Smartphone-based patient-reported mood^b^	**−1.37**	**−2.00;** **−0.73**	** <0.001**

a *Smartphone-based patient-reported activity rated on a 7-point scale from −3 to +3*.

b *Smartphone-based patient-reported mood rated on a 5-point scale from −3 to 0*.

*n = number of unique patients in the analysis o = number of days with patient-reported data and corresponding automatically generated data in the analysis*.

*Bold text reflects statistically significant findings with p <0.05*.

#### Smartphone-Based Patient-Reported Mood

As hypothesized, smartphone-based patient-reported mood was negatively associated with automatically generated measures of social activity and phone usage: Lower patient-reported mood was associated with higher automatically generated measures. [Adjusted models: screen time: B = −1343.6, 95% CI (−2196.93; −490.26), *p* = 0.0020; call duration: B = −256.81, 95% CI (−370.13; −143.50), *p* < 0.001; the number of incoming calls: B = −0.30, 95% CI (−0.46; −0.14), *p* < 0.001; the number of missed calls: B = −0.17, 95% CI (−0.31; −0.028), *p* = 0.019; the number of incoming text messages: B = −1.87, 95% CI (−2.54; −1.20), *p* < 0.001 and the number of outgoing text messages: B = −1.37, 95% CI (−2.00; −0.73), *p* < 0.001]. Thus, for every decrease of patient-reported mood by one point, there was an increase of daily screen time by 1,344 s (22 min).

#### Smartphone-Based Patient-Reported Activity

As hypothesized, smartphone-based patient-reported activity was positively associated with automatically generated measures of physical and social activity as well as phone usage: Lower patient-reported activity was associated with a decrease in [adjusted models: number of steps: B = 178.58, 95% CI (32.61; 324.54), *p* = 0.017; distance traveled: B = 4.948, 64.95% CI (2239.70; 7647.58), *p* < 0.001, number of times screen is turned on: B = 1.32, 95% CI (0.45; 2.19), *p* = 0.0030 and number of missed calls: B = 0.085, 95% CI (0.027; 0.14), *p* = 0.0041]. Further, Smartphone-based patient-reported activity was negatively associated with screen time and number of incoming calls: Lower patient-reported activity was associated with an increase of (adjusted models: Total screen time: B = −783.63, 95% CI (−1122.90; 444.36), *p* < 0.001; and the number of incoming calls: B = −0.088; 95% CI (−0.15; −0.023), *p* = 0.0082.) Thus, for every decrease in patient-reported activity by one point, there was a corresponding decrease in the daily length traveled by 4,949 m and an increase in the daily screen time by 784 s (13 min).

## Discussion

We systematically investigated smartphone-based monitoring tools for patients with unipolar depressive disorder against validated clinical ratings and questionnaires.

### Associations Between Patient-Reported Mood and Activity, Respectively, and Validated Ratings and Questionnaires (Aim 1)

As hypothesized, smartphone-based patient-reported mood and activity were associated with total scores on the HDRS-17 and the FAST. Further, smartphone-based patient-reported mood was statistically significantly associated with mood according to item 1 of the HDRS-17 and with the BDI-21. These findings emphasize that patient-reported mood and activity are feasible and with high clinical validity in patients with unipolar depressive disorder. This is consistent with similar studies on patients with bipolar disorder (14, 15, 29). In this way, smartphone-based daily patient-report allows for unobtrusive, fine-grained monitoring of core symptoms of illness activity and can be used for outpatient monitoring.

### Associations Between Automatically Generated Smartphone Measures and Clinical Ratings (Aim 2)

The associations of clinical ratings of depression (including subscores) with behavioral changes detected through automatically generated smartphone data were not as compelling as expected. We found four statistically significant results among 30 analyses, with a high risk of chance findings: As hypothesized, higher HDRS-17 total scores were associated with a higher number of times the screen was turned on and longer duration of phone calls, whereas, higher scores on HDRS item 9 (agitation) were associated with more outgoing calls and longer duration of phone calls.

Lower levels of psychosocial functioning resulted in fewer daily steps, an increased number of incoming, outgoing, and missed calls, and increased duration of phone calls. Thus, several detectable changes in behavior were found to be associated with the FAST. However, the increase of phone-call-activity possibly represents an increased concern and help from family, friends, or caretakers, rather than changes in symptoms.

The recent technological development has shifted our digital life toward smartphones (41), providing a wealth of data with high ecological validity. However, the interpretation of automatically generated EMA is not as straightforward as the patient-reported EMA, and changes in technology behavior might alter interpretations. Furthermore, instead of looking at the individual measures one by one, techniques for creating clinically relevant composite scores might be useful for future studies (30, 42).

### Associations Between Smartphone-Based Patient-Reported Mood and Activity and Automatically Generated Smartphone Data (Aim 3)

As hypothesized, when combining the daily smartphone-based patient-reported data with the same-day automatically generated smartphone data, we found that patient-reported mood and activity were associated with several changes in behavior reflected by automatically generated smartphone data. The changes were in line with our hypotheses, findings from previous studies, and clinical experience.Thus, smartphone usage could be a proxy measure for sedentary behavior. Lower patient-reported mood was associated with an increase in smartphone usage (as a possible sign of more sedentary behavior) and an increase in missed calls as a possible sign of less social engagement. The prolonged call duration could be caused by the increase in incoming calls. Along with an increase of incoming text messages, the increased incoming communication may reflect increased concern/care from surroundings when patients report low mood.

Lower smartphone-based patient-reported activity was associated with a decrease in the number of steps and distance traveled as a sign of lower physical- and travel activity. As with patient-reported mood, the corresponding increase of total screen time, the number of missed and incoming calls may reflect an increase in sedentary behavior, social withdrawing, and concern from surroundings, respectively.

As such, same-day patient-reported mood and activity were associated with specific behavioral changes. Thus, a large amount of non-intrusive, fine-grained same-day smartphone data can be collected and may reflect changes in symptoms and behavior caused by the severity of the disease.

### Limitations

The data in the present study is obtained from an RCT study. Participation in the study might have affected the patients' smartphone behavior when planning and showing up for assessments. Further, automatically generated smartphone data may have been influenced by the intervention to some degree as patients were contacted on text message or telephone by the study-nurse in case of concern. Due to different technological and methodological reasons, not all data was collected from all patients. There are constant technological changes to smartphone technology and to how we use and interact with our smartphones. These changes may influence the future generalizability of our findings, especially concerning the type of data collected and how it is processed. We only collected data on old school text messages, not including common messenger apps or social media. Future studies may include more broad sources of smartphone-based information (21). The fact that the data were collected in the period following discharge might influence the generalizability of the findings. In the present study we did not employ non-linear statistical models. Future studies employing non-linear time-series analyses could provide more in insights into this area.

### Advantages

The combination of smartphone-based technology and thorough clinical assessments in a clinical population is a major advantage of the present study. Rating scales were conducted by research-trained medical doctors in well-tested clinical setups and with high attrition among included patients. Patients underwent thorough clinical examinations, and researchers had access to their electronic medical records. The data were collected in a population of patients with an indisputably need for psychiatric support and treatment, and thus, findings from the current study can more easily be generalized to patient populations in psychiatric treatment settings. Finally, the system used in the study had been thoroughly tested in multiple studies in patients with bipolar disorder (14, 29, 33, 43, 44).

### Perspective

The ongoing COVID-19 crisis, with worldwide and local lock-down(s), has accelerated and emphasized the need for new ways of conducting outpatient psychiatry assisted by technology. Smartphone-based solutions could likely improve telephone and online courses by supplying clinicians with important information about patients' symptoms and behavior. Moreover, methods to adapt to the constant technological changes to smartphone technology faster are needed. Machine-learning algorithms might detect such patterns (45), and techniques developed in recent research (25, 46) might be used to evaluate and adapt on an individual basis (applying machine learning user dependent models) rather than focusing on group statistics.

### Conclusion

Smartphone-based symptom monitoring in patients with unipolar depressive disorder was feasible and associated with clinical ratings of depression and psychosocial functioning. Patient-reported mood and activity were highly statistically significantly associated with standardized clinical ratings and questionnaires. The results concerning the association of automatically generated smartphone data with clinical ratings of depression were less appealing, and only 4/30 associations were statistically significant. Lower levels of clinically rated psychosocial functioning were associated with fewer daily steps, increased number of incoming, outgoing, and missed calls, and increased duration of phone calls. Finally, the associations of smartphone-based patient reportings and same-day automatically generated smartphone data were more consistent and showed that the daily patient reportings were associated with several automatically generated smartphone features likely reflecting behavioral changes. Taken together, findings from this study suggest that smartphone-based patient-reported data on mood and activity and some smartphone-based automatically generated data on behavior may assist and supplement the clinicians in the monitoring of unipolar depressive disorder, and hereby provide patients and healthcare professionals relevant and timely information to improve decision making and treatment. A combination of data could potentially increase the use of smartphone data in clinical settings and should be investigated further using more advanced machine learning models in future studies, i.e., focusing on individual patterns and generating composite scores.

## Data Availability Statement

The datasets presented in this article are not readily available because data are still being used for internal research purposes, and we do not have the approvals for data sharing. Requests to access the datasets should be directed to Lars.Vedel.Kessing@regionh.dk.

## Ethics Statement

The studies involving human participants were reviewed and approved by the ethics committee in the Capital Region of Denmark (H-16046093). The patients/participants provided their written informed consent to participate in this study.

## Author Contributions

MT, MF-J, JB, and LK conceived the study. MT, MF-J, and LK did all statistical analyses and wrote the first draft of the manuscript. JB and MF provided the technical content. All authors contributed to the manuscript and approved the final version.

## Conflict of Interest

JB and MF are co-founders and shareholders of Monsenso. LK has, within 3 years, been a consultant for Lundbeck and Teva. The remaining authors declare that the research was conducted in the absence of any commercial or financial relationships that could be construed as a potential conflict of interest.
